# Expression and clinical significance of CCN5 and the oestrogen receptor in advanced breast cancer

**DOI:** 10.1186/s12905-025-03608-3

**Published:** 2025-02-27

**Authors:** Guofeng Zhou, Wei Qu, Liu Yang, Aili Huang, Xingxing Gui

**Affiliations:** Department of Pathology, Nanchang People’S Hospital (the Third Hospital of Nanchang), No.1268, Jiuzhou Street, Xihu District, Nanchang, 330009 China

**Keywords:** Breast cancer, CCN5, Estrogen receptor, Ductal carcinoma in situ, Invasive carcinoma

## Abstract

**Purpose:**

The aim of this study was to investigate the expression and clinical implications of CCN family member 5 (CCN5) and the oestrogen receptor (ER) in advanced breast cancer (BC).

**Methods:**

A total of 130 patients with advanced BC were selected for the study. Samples of normal breast tissue, ductal carcinoma in situ (DCIS), and invasive carcinoma were collected. The expression levels of CCN5 and ER in these tissues were examined using immunohistochemical methods. The correlation between expression of CCN5 and ER in different tissues and also differences in expression in invasive carcinoma were analysed. In addition, the relationship between CCN5 expression in advanced BC tissues and clinical pathological features was examined.

**Results:**

CCN5 and ER had low expression in normal breast tissues and invasive carcinoma tissues, but high expression in DCIS, with this difference being statistically significant (X^2^ = 119.899, *P* < 0.001; X^2^ = 113.524, *P* < 0.001, respectively). The expression of CCN5 and ER in different tissues of patients with advanced BC showed a positive correlation. Significant differences were also observed in the positive and negative expression of CCN5 and ER (X^2^ = 56.358, *P* < 0.001). Moreover, the expression of CCN5 protein in advanced BC showed a statistically significant associations (*P* < 0.05) with the expression of the progesterone receptor (PR), human epidermal growth factor receptor 2 (HER-2), Ki-67, and P53, tumor diameter, histological grade, lymph node metastasis, pathological molecular subtype, and clinical staging.

**Conclusion:**

High expression of CCN5 and ER was observed in DCIS tissues of patients with advanced BC, with their expression being positively correlated. These findings suggest that CCN5 and ER may have a potential synergistic role in the progression of BC that influences the progression of advanced BC and can also be used to predict the effectiveness of endocrine therapy.

## Introduction

Breast cancer (BC) is the most common malignant tumour in women worldwide [1]. However, Advanced breast cancer, comprising the most serious stages, stage 3 (locally advanced disease) and stage 4 (metastatic stage) of breast cancer, is generally considered as a treatable but still incurable disease because of its high rates of recurrence and resistance to chemotherapy [2].The oestrogen receptor (ER) is a member of the nuclear receptor superfamily and is a ligand-dependent transcription factor that is used as a crucial parameter for predicting the survival of BC patients and also a key indicator for planning personalized treatment plans [3, 4]. ER-positive BC accounts for 75% of all BC cases. This type of BC is treated primarily using anti-hormonal therapy, with current medications including tamoxifen, an aromatase inhibitor or fulvestrant [5, 6]. Despite significant breakthroughs in treatment efficacy, resistance to endocrine therapy is quite common, especially in ER-positive advanced BC, which remains incurable [7–9]. Dysregulation, dysfunction, or inhibition of ER-α has been linked to tumour aggressiveness, metastasis, and potential hormone resistance [10–12]. In addition, CCN family member 5 (CCN5) plays a crucial role in maintaining the differentiated phenotype of ER-positive BC [13], with the absence of CCN5 associated with epithelial-mesenchymal transition (EMT) [14–16]. Based on these findings, BC research has focused on CCN5 and ER.

CCN5, also known as Wnt-induced secreted protein (WISP-2), is a new member of the CCN family and plays a significant role in the regulation of mitosis, cell adhesion, induction of apoptosis, extracellular matrix production, growth inhibition, and the migration of various cells [17, 18]. CCN5 protein is a tumour suppressor gene and plays a crucial role in the onset, progression, and aggressiveness of BC [19, 20]. Studies have shown that the expression of CCN5 is inversely correlated with the aggressiveness of breast cancer, indicating its oncogenic/anti-invasive activity [18, 20, 21]. Therefore, in BC, CCN5 can be considered as a good prognostic marker [18, 21]. BC cells with CCN5 deletion expression were less invasive compared to BC cells with insufficient or negative CCN5 expression [18, 22, 23]. Mechanistically, multiple genetic insults, including acquisition of p53 mutations, deplete CCN5 expression at the transcriptional level in non-aggressive BC cells and help the cells acquire an aggressive phenotype [24]. CCN5 regulation in ER-positive BC cells is dependent on oestrogen, insulin-like growth factor, and HIF-α2, the expression of which has been found to be involved in the control of the proliferative and invasive phenotype of these cells [14, 18, 25]. Thus, depletion of CCN5 in ER-positive BC cells promotes oestrogen-dependent growth, EMT, and stemness, consistent with a more aggressive phenotype. CCN5 expression may be an early genetic event closely related to the development of BC, and also serves as a marker of potential progression in BC, typically in ER-positive cases [26]. In ER-negative BC, ectopic expression of CCN5 enhances ER expression [27]. Therefore, it is necessary to elucidate the interactive relationship and clinical significance of CCN5 and ER in the progression of BC.

Given the complex interactions between CCN5, ER, and progression of BC, the aim of the current study was to analyse the expression of CCN5 and ER in normal breast tissue, ductal carcinoma in situ (DCIS), and invasive carcinoma tissues of patients with advanced BC. The correlation between CCN5 and ER expressions in different tissues and their differential expression in invasive carcinoma were also examined. The study also investigated the relationship between CCN5 expression in advanced BC tissues and clinical pathological parameters in order to determine the impact of CCN5 and ER on the development and progression of advanced BC. This information provided valuable insights into the mechanisms involved in the progression of BC. Here, we hypothesized that CCN5 and ER are highly expressed and positively correlated in DCIS tissues from patients with advanced breast cancer, suggesting that CCN5 and ER may act synergistically to influence advanced breast cancer progression during breast cancer development, providing valuable insights for future research and clinical practice.

## Methods

### General information

**Fig. 1:**
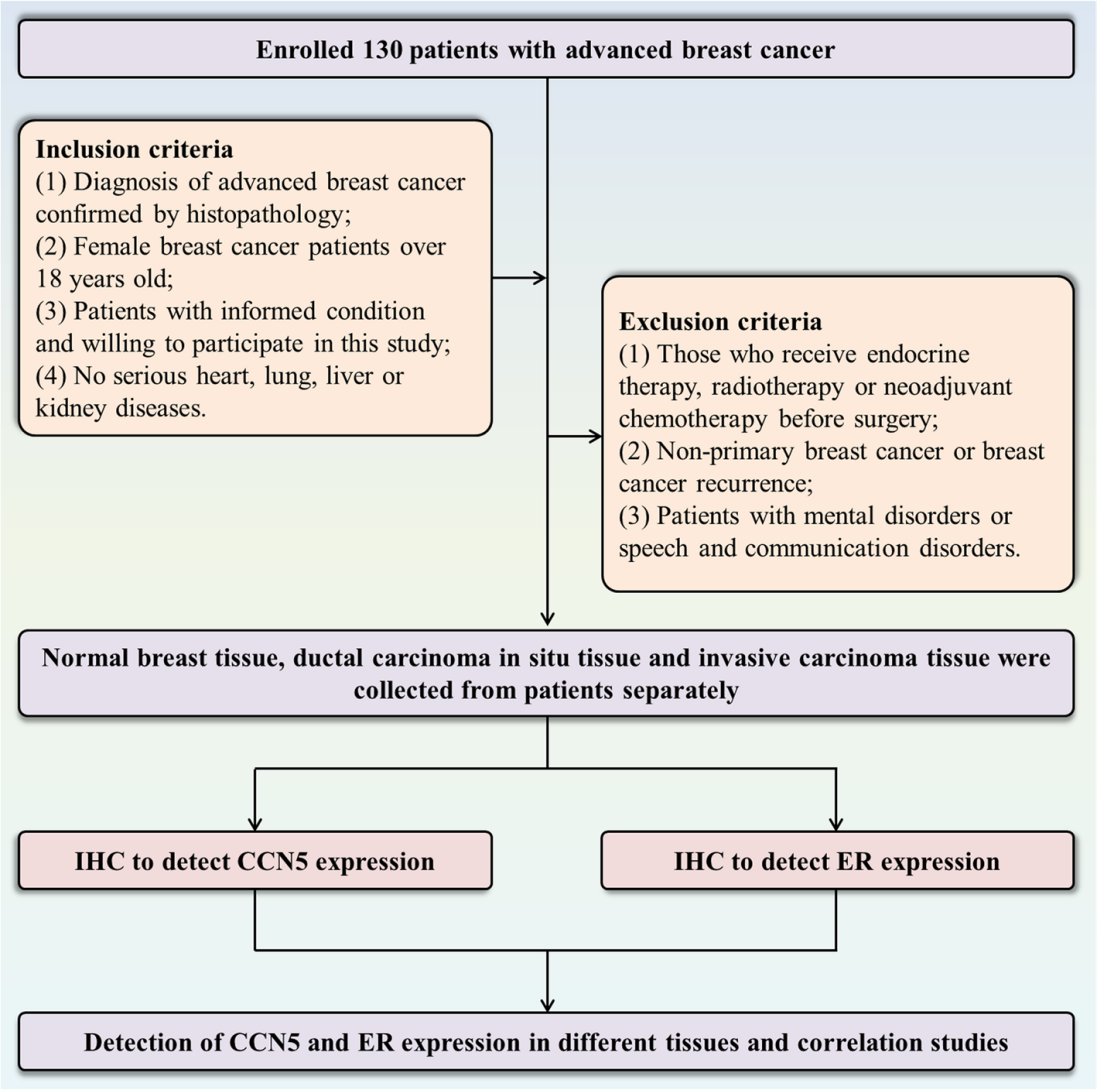
Schematic diagram of the research workflow

Patients with advanced breast cancer between January 2022 and December 2023 were screened from Nanchang People’s Hospital (The Third Hospital of Nanchang), and the clinicopathological information of the 130 patients with advanced breast cancer was obtained from the electronic medical record system of our hospital, including the age at diagnosis, histopathology, and TNM stage, etc. Each patient’s sample included paired normal breast tissue, DCIS tissue, and invasive carcinoma tissue. All the patients were female. The pathological diagnoses were confirmed by two or more senior pathologists from our hospital’s clinical pathology department. The patients all had complete clinical and pathological data. Inclusion criteria: (1) Histopathologically confirmed advanced BC, (2) female BC patients aged older than 18 years, (3) patients informed of their condition and agreed to participate in the study, (4) no history of severe cardiac, pulmonary, hepatic, or renal diseases. Exclusion criteria: (1) Patients who received either endocrine therapy, radiation therapy, or neoadjuvant chemotherapy prior to surgery, (2) non-primary BC or recurrent BC, (3) patients with psychiatric disorders or communication impairments (Fig. 1).

### Materials and reagents

Concentrated rabbit anti-human polyclonal CCN5 was purchased from OriGene Technologies, USA (catalogue number, TA36806S). ER (catalogue number, AR0710), progesterone receptor (PR) (catalogue number, AR0711), Ki-67 (catalogue number, AR0248), and P53 (catalogue number, AM0183) were all acquired from Xiamen Talent Biomedical Technology Co., Ltd. Human epidermal growth factor receptor 2 (HER-2) (clone number, 4B5) was sourced from Roche Diagnostics (Shanghai) Ltd. Secondary antibody detection reagent was obtained from Fuzhou Maixin Biotech, Ltd. (catalogue number: KIT-5053), diaminobenzidine (DAB) from Shanghai Liangrun Biomedicine Technology Co., Ltd (catalogue number: SD3001), and fluorescence in situ hybridization (FISH) test kits from Wuhan HealthCare Biotechnology Co., Ltd. (catalogue number: FP-001).

### Immunohistochemical analysis

Immunohistochemistry was conducted using the streptavidin–biotin (SP) method, according to the detailed steps provided in the reagent manual. Phosphate-buffered saline (PBS) was used as the negative control instead of the primary antibody, while known BC positive slides were used as the positive control. The main steps in the method were: All specimens were fixed in 10% neutral buffered formalin; routinely dehydrated; embedded in clear paraffin; sectioned into 4 μm thick consecutive slices; dewaxed in xylene, blocked with H_2_O_2_; antigen retrieval by heating; incubation with primary antibody overnight at 4 °C; secondary antibody incubation for 30 min; development with DAB; counterstained with haematoxylin; and cover-slipped with neutral balsam.

### Interpretation of the immunohistochemical results

(1) Positive expression of ER, PR Refering to the Version 2020 ASCO / CAP guide was defined as ≥ 1% of tumour cells showing varying degrees of nuclear and cytoplasmic staining. (2) Negative expression of ER, PR was defined as < 1% of tumour cells showing varying staining or no staining. (3) The positive expression of CCN5 protein was mainly the appearance of brownish yellow particles in the cytoplasm. The staining results were observed by double-blind method, and the color intensity and the proportion of positive cells were comprehensively evaluated comprehensively [28]. No positive coloring is 0 points, light yellow is one point, brown yellow is two points, and brown is three points [29]. Ten high-power fields were randomly selected, and the percentage of positive cells in every 200 cells was estimated for each field. An average was taken for the 10 fields. The scoring scale utilized in this study to assess CCN5 and expression levels was adapted from previous research [30]. This scoring system assigns points based on staining intensity and the percentage of positive cells, with a total score ≤ 3 considered negative and a total score > 3 considered positive. The utilization of a standardized scoring scale in this study allowed for the objective assessment of CCN5 expression levels in breast cancer tissues [30]. (4) Interpretation of HER-2 testing followed the Chinese Guidelines for Detection of HER-2 in Breast Cancer (2019 edition), with positive staining defined as > 10% of invasive cancer cells having strong and complete membrane staining (3 +), and negative staining as 1 + and 0. (5) The Ki-67 proliferation index According to the consensus of the International Expert Group of Breast Cancer was calculated as high expression (≥ 20% of positive cells) or low expression (< 20% of positive cells). (6) p53 was classified into wild-type or mutant types according to the staining pattern, which mainly relies on the PROMISE molecular typing method proposed by the Canadian scholar Mcconechy et al. [28].

### FISH testing

Cases with a HER-2 immunohistochemical scoring of 2 + underwent FISH testing. The procedure was carried out using a FISH test kit, following the instructions provided in the kit. The cases were categorized into HER-2 negative and positive groups based on the presence or absence of HER-2 gene amplification.

### Statistical analysis

Statistical analysis was performed using SPSS 21.0 software (IBM Corp., Armonk, NY, USA). Non-parametric tests, especially chi-square tests, were used to compare the expression levels of CCN5 and ER in different tissues. To assess the correlation between CCN5 and ER expression levels in breast tissues, Spearman rank correlation analysis was used. All statistical tests were two-sided, and a difference of *P* < 0.05 was considered statistically significant.

## Results

### Clinical characteristics

The study included 130 BC patients aged between 27 and 84 years, with a mean age of 54 years. A total of 53 cases involved the left breast and 77 the right breast, with the tumour sizes ranging from 1.2 to 12 cm (mean size 4.82 cm). The patients were staged according to the 8th edition of the American Joint Committee on Cancer (AJCC) clinical pathological tumour node metastasis (pTNM) staging system for advanced BC: stage IIB in 14 cases (10.8%), IIIA in 25 cases (19.23%), IIIB in 12 cases (9.23%), and IIIC in 79 cases (60.77%).

### Analysis of CCN5 and ER expression

**Fig. 2:**
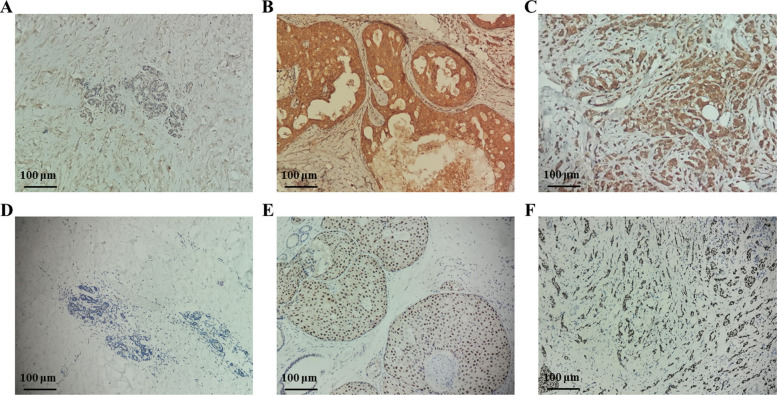
Immunohistochemical findings in normal breast tissue and breast tumour tissue. **A** CCN5 expression in normal breast tissue (× 100); **B** CCN5 expression in breast DCIS (× 100); **C** CCN5 expression in breast invasive carcinoma (× 100); **D** ER expression in normal breast tissue (× 100); **E** ER expression in breast DCIS (× 100); **F** ER expression in breast invasive carcinoma (× 100)

CCN5 and ER were expressed in the cytoplasm and nuclei of the BC cells (Figs. 2A-F). CCN5 and ER showed low expression in normal breast tissue and invasive carcinoma tissue, but high expression in DCIS tissue. As shown in Table 1, the differences in expression were statistically significant (X^2^ = 119.899, *P* < 0.001; X^2^ = 113.524, *P* < 0.001).

**Table 1:** Expression of CCN5 and ER in normal breast tissue, DCIS tissue, and invasive carcinoma tissue [n (%)]

Tissue type	CCN5		X^2^	*P*-value	ER		X^2^	*P*-value
	Positive (%)	Negative (%)			Positive (%)	Negative (%)		
Normal breast tissue	16 (12.30)	114 (87.69)	119.899	< 0.001	14 (10.77)	116 (89.23)	113.524	< 0.001
DCIS tissue	103 (79.23)	27(20.77)			98 (75.38)	32 (24.62)		
Invasive carcinoma tissue	51 (39.23)	79 (60.77)			48 (36.92)	82 (63.08)		

### Correlation analysis of CCN5 and ER expression in normal breast tissue, DCIS tissue, and invasive carcinoma tissue

The expression of CCN5 and ER in BC showed a positive correlation in normal breast tissue (rs = 0.014, *P* = 0.827), DCIS tissue (rs = 0.046, *P* = 0.461), and invasive carcinoma tissue (rs = 0.024, *P* = 0.703) (Table 2).

**Table 2:** Correlation between CCN5 and ER expression in normal breast tissue, DCIS tissue, and invasive carcinoma tissue (*n* = 130)

Tissue type	CCN5		ER		rs	*P*-value
	Positive	Negative	Positive	Negative		
Normal breast tissue	16	114	14	116	0.014	0.827
DCIS tissue	103	27	98	32	0.046	0.461
Invasive carcinoma tissue	51	79	48	82	0.024	0.703

### Expression of CCN5 and ER in invasive carcinoma of advanced BC

In advanced BC, the positive expression rates of CCN5 and ER in invasive carcinoma were 39.22% (51/130) and 36.92% (48/130) respectively, while the negative expression rates were 60.77% (79/130) and 63.08% (82/130) respectively. The differences in positive and negative expression of CCN5 and ER were statistically significant (X^2^ = 56.358, *P* < 0.001) (Table 3).

**Table 3:** Expression of CCN5 and ER in invasive carcinoma [n (%)]

ER	CCN5		Total	X^2^	OR (95%CI)	*P*-value
	Positive	Negative				
Positive	39 (71.25)	9 (18.75)	48	56.358	25.2 (9.8—65.3)	< 0.001
Negative	12 (14.63)	70 (85.37)	82			
Total	51 (39.23)	79 (60.77)	130			

### Association between CCN5 expression and clinicopathological features in advanced BC

**Fig. 3:**
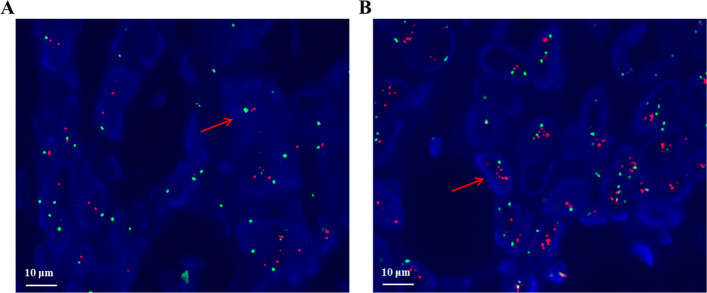
FISH results of HER-2 negative and HER-2 positive samples. **A** HER-2 results of HER-2 negative samples (× 400); **B** HER-2 results of HER-2 positive samples (× 400)

In advanced BC, the expression of CCN5 protein correlated significantly (*P* < 0.05) with PR, HER-2 (Fig. 3), Ki-67, P53, tumour diameter, histological grade, lymph node metastasis, pathological molecular subtype, and clinical staging. However, there were no statistically significant differences in expression related to age or the site of the tumour (*P* > 0.05) (Table 4).

**Table 4:** Relationship between CCN5 expression and clinicopathological features in 130 cases of advanced BC [n (%)]

Clinicopathological parameter	n	CCN5		X^2^	OR (95%CI)	*P*-value
		Positive	Negative			
PR				26.096	7.9 (3.4—18.2)	< 0.001
Positive	46	33 (71.74)	13 (28.26)			
Negative	74	18 (24.32)	56 (75.68)			
HER-2				14.928	5.3 (2.2—12.9)	< 0.001
Positive	28	19 (67.86)	9 (32.14)			
Negative	112	32 (28.57)	80 (71.43)			
Ki-67				45.489	0.1 (0—0.1)	< 0.001
High expression	84	15 (17.86)	69 (82.14)			
Low expression	46	36 (78.26)	10 (21.74)			
P53				33.392	12.5 (4.8—32.4)	< 0.001
Wild type	35	28 (80.00)	7 (20.00)			
Mutant type	95	23 (24.21)	72 (75.89)			
Age (years)				0.344	1.2 (0.6—2.5)	0.557
< 54	52	22 (42.31)	30 (57.69)			
≥ .5	78	29 (37.18)	49 (62.82)			
Breast side				1.375	1.5 (0.7—3.1)	0.241
Left breast	53	24 (45.28)	29 (54.72)			
Right breast	77	27 (35.06)	50 (64.94)			
Tumour diameter (cm)				11.390		0.003
< 2	8	6 (75.00)	2 (25.00)		1 (0.1—9.6)	
2–5	65	31 (47.69)	34 (52.31)		1 (0.5—2.0)	
> 5	57	14 (24.56)	43 (75.44)		1 (0.4—2.3)	
Lymph node metastasis				7.225	0.3 (0.1—0.8)	0.007
Yes	98	32 (32.65)	66 (67.35)			
No	32	19 (59.38)	13 (40.62)			
Histological grade				3.983	2.4 (1.0—5.7)	0.046
II	94	42 (44.68)	52 (55.32)			
III	36	9 (25.00)	27 (75.00)			
Pathological molecular subtype				16.450		< 0.001
Luminal A	55	31 (56.36)	24 (43.64)		1 (0.5—2.1)	
Luminal B	26	11 (42.31)	15 (57.69)		1 (0.3—3.0)	
HER-2 overexpression	31	7 (22.58)	24 (77.42)		1 (0.3—3.3)	
Triple-negative	18	2 (11.11)	16 (88.89)		1 (0.1—8.0)	
Clinical staging				37.787		< 0.001
IIB	14	10 (71.43)	4 (28.57)		1 (0.2—5.2)	
IIIA	25	17 (68.00)	8 (32.00)		1 (0.3—3.3)	
IIIB	12	9 (75.00)	3 (25.00)		1 (0.2—6.3)	
IIIC	79	15 (18.99)	64 (81.01)		1 (0.5—2.2)	

## Discussion

CCN5 (also known as WISP-2, HICP, rCOP1, or CTGF-L) is a 29 kD secretory protein and a member of the CCN family that also functions as a tumour suppressor gene. CCN5 shares 30–40% sequence homology with other members of the CCN family, but lacks the cysteine knot domain. It plays a significant role in regulating cellular proliferation, motility, invasiveness, and adhesiveness [29, 30]. CCN5 is of critical importance in the development of BC, as it exhibits differential expression in BC cell lines and BC tissue samples [30–32]. CCN5 is present predominantly in non-invasive BC cell lines and pre-malignant breast lesions, with higher expression than that observed in normal breast tissues. However, its expression decreases in invasive BC and in adjacent invasive foci, with the level of CCN5 also decreasing as tumour differentiation worsens, leading to very low expression in poorly differentiated tumours [4]. It has been shown that the absence of CCN5 drivers in BC promotes cancer cell growth and EMT, whereas up-regulation of CCN5 is associated with ER activation in normal and cancerous cells in human and mouse mammary glands [33, 34]. Research has also shown that a decline in CCN5 expression in BC is associated with ER transcription levels [18]. In this study on 130 cases of advanced BC, CCN5 and ER were expressed in normal breast tissue, DCIS tissue, and invasive carcinoma tissue, with CCN5 showing a low–high-low expression pattern. This indicated that CCN5 has a role as a tumour suppressor gene. As the breast tumour progresses, the expression level of CCN5 decreases gradually, suggesting it has a role in inhibiting the transition of breast tumour epithelium from non-invasive to invasive stages. In addition, the expression rate of ER also decreases, indicating that ER progressively becomes inactive as BC progresses. Therefore, the decline in expression of both CCN5 and ER has predictive significance for the prognosis of patients with advanced BC and provides a measure on the effectiveness of endocrine therapy.

Research indicates that over 60% of BC patients have an immunological response to the ER-α antibody. ER-α is a crucial biomarker and prognostic indicator that suggests lower aggressiveness of BC and responsiveness to endocrine treatments such as tamoxifen [35, 36]. However, only two-thirds of late-stage ER-positive BC respond effectively to endocrine therapy [4, 8]. Non-responsive tumours may not express ER-α or may initially be positive but lose function during aggressive growth, potentially becoming hormone-independent and resistant to endocrine therapy, eventually leading to a loss of ER-α expression [26]. It has been shown that CCN5, by interacting with integrin-α6β1, inhibiting Akt, and then activating FOXO3a, is sufficient to induce ER-αexpression at the transcriptional level [4]. Therefore, the absence of ER-α expression in BC is a major factor that contributes to the aggressiveness and recurrence of disease [37]. However, the mechanisms regulating the expression and activity of ER-α in normal breast tissue and BC are not well understood. Research by Dhar et al. [38] reported that in normal mammary epithelial or breast tumour cells, inducing the expression of CCN5 or treating with CCN5 protein significantly increases ER-α expression. Conversely, inhibiting CCN5 through shRNA or antibody treatment reduces ER-α expression in ER-α positive BC cells. The findings of these studies also suggested that upregulated ER-α maintains functional activity in both normal and transformed cells. CCN5 is therefore an oestrogen-responsive gene in ER-α positive BC cells [34, 39]. Treatment with estradiol activates synthetically produced CCN5 (hrCCN5), possibly through autocrine-paracrine feedback signalling loop involving CCN5 and ER-α that regulates these molecules, and activates CCN5 transcription in triple-negative BC [40].

The current study showed a positive correlation in the expression of CCN5 and ER across normal breast tissue, DCIS tissue, and invasive carcinoma tissue. These findings indicate that CCN5 and ER have a synergistic role in the development of BC and may potentially inhibit the progression of breast tumours. The high expression of CCN5 in ER-positive advanced BC compared to the low or negative expression of CCN5 in ER-negative advanced BC, suggests an effective regulatory interaction exists between the CCN5 signalling pathway and the ER pathway in BC [36]. Consistent with the findings of our study, previous studies [40] have also reported that BCs with high CCN5 expression are less aggressive than those with low or negative CCN5 expression. In addition, the expression rate of PR gradually decreases in association with a variety of changes that include increased HER-2 positivity, elevated Ki67 index, the emergence of P53 mutations, larger tumour diameters, lymph node metastasis, higher histological grades, the occurrence of triple-negative BC in pathological molecular subtypes, and an advancement in clinical staging. These changes result in a progressive decline in the expression rate of CCN5 and indicate that advanced BC is more aggressive and has a poorer prognosis.

Although CCN5 has been widely studied on breast cancer. However, CCN5 has been poorly studied on ER positive advanced breast cancer. There are some limitations of this study, firstly, the sample is small and retrospective, further studies with larger and more homogeneous cohorts and standardized assessment methods are needed to clarify the prognostic significance of these markers. Secondly, there is a lack of some detailed clinical data, such as biological characteristics, which could further reveal the effects of these factors on CCN5 and ER expression levels. Finally, the mechanism of how CCN5 regulates ER-positive advanced breast cancer is not clear. This mechanism has the potential to find that CCN5 improves the efficacy of ER positive advanced breast cancer and improves the patient survival rate.

## Conclusion

CCN5 and ER are highly expressed in DCIS tissues of patients with advanced BC, and their expression is positively correlated. This suggests that CCN5 and ER may have a potential synergistic role in the progression of BC, contributing to inhibition of the disease’s advancement. Furthermore, CCN5 guides the endocrine treatment and prognosis of ER-positive advanced BC and therefore has significant clinical implications.

## Data Availability

The data that support the findings of this study are not openly available due to reasons of sensitivity and are available from the corresponding author upon reasonable request.

## References

[1] Katsura C, Ogunmwonyi I, Kankam HK, et al. Breast cancer: presentation, investigation and management. Br Hosp Med (Lond). 2022;83(2):1–7. 10.12968/hmed.2021.0459 35243878

[2] Liedtke C, Kolberg HC. Systemic therapy of advanced/metastatic breast cancer—current evidence and future concepts. Breast Care. 2016;11:275–81. 27721716 10.1159/000447549 PMC5040928

[3] Garcia-Martinez L, Zhang Y, Nakata Y, et al. Epigenetic mechanisms in breast cancer therapy and resistance. Nat Commun. 2021;12(1):1–14. 33741974 10.1038/s41467-021-22024-3 PMC7979820

[4] Giacinti L, Claudio PP, Lopez M, et al. Epigenetic information and estrogen receptor alpha expression in breast cancer. Oncologist. 2006;11(1):1–8. 16401708 10.1634/theoncologist.11-1-1

[5] Clarke R, Skaar T, Baumann K, et al. Hormonal carcinogenesis in breast cancer: cellular and molecular studies of malignant progression. Breast Cancer Res Treat. 1994;31(2–3):237–48. 7881102 10.1007/BF00666157

[6] Harbeck N, Penault-Llorca F, Cortes J, et al. Breast cancer. Nat Rev Dis Primers. 2009;5(1):66. 10.1038/s41572-019-0111-2 31548545

[7] Harrell JC, Dye WW, Allred DC, et al. Estrogen receptor positive breast cancer metastasis: altered hormonal sensitivity and tumor aggressiveness in lymphatic vessels and lymph nodes. Cancer Res. 2006;66:9308–15. 16982776 10.1158/0008-5472.CAN-06-1769

[8] Hartkopf AD, Grischke EM, Brucker SY. Endocrine-resistant breast cancer: mechanisms and treatment. Breast Care (Basel). 2020;15(4):347–54. 32982644 10.1159/000508675 PMC7490658

[9] Osborne CK, Schiff R. Mechanisms of endocrine resistance in breast cancer. Ann Rev Med. 2011;62(1):233–47. 20887199 10.1146/annurev-med-070909-182917 PMC3656649

[10] Lacroix M, Leclercq G. Relevance of breast cancer cell lines as models for breast tumours: an update. Breast Cancer Res Treat. 2004;83:249–89. 14758095 10.1023/B:BREA.0000014042.54925.cc

[11] Vesuna F, Lisok A, Kimble B, Domek J, Kato Y, van der Groep P, et al. Twist contributes to hormone resistance in breast cancer by downregulating estrogen receptor-alpha. Oncogene. 2012;31:3223–34. 22056872 10.1038/onc.2011.483 PMC3276743

[12] Murphy CG, Dickler MN. Endocrine resistance in hormone-responsive breast cancer: mechanisms and therapeutic strategies. Endocr Relat Cancer. 2016;23(8):R337-352. 27406875 10.1530/ERC-16-0121

[13] Ferrand N, Gnanapragasam A, Dorothee G, et al. Loss of WISP/CCN5 in estrogen-dependent MCF7 human breast cancer cells promotes a stem like cell phenotype. PLoS ONE. 2014;9(2): e87878. 24498388 10.1371/journal.pone.0087878 PMC3912128

[14] Das A, Dhar K, Maity G, et al. Deficiency of CCN5/WISP-2 driven program in breast cancer promotes cancer epithelial cells to mesenchymal stem cells and breast cancer growth. Sci Rep. 2017;7(1):1220. 28450698 10.1038/s41598-017-00916-z PMC5430628

[15] Zhang T, Han J. Research progress of epithelial-mesenchymal transition in breast cancer. Chongq Medic. 2016;45(33):4722–4.

[16] Roxanis I. Occurrence and significance of epithelial-mesenchymal transition in breast cancer. J Clin Pathol. 2013;66(6):517–21. 23322823 10.1136/jclinpath-2012-201348

[17] Barreto SC, Ray A, Ag EP. Biological characteristics of CCN proteins in tumor development. J Buon. 2016;21(6):1359–67. 28039692

[18] Banerjee SK, Banerjee S. CCN5/WISP-2: a micromanager of breast cancer progression. J Cell Commun Signal. 2012;6:63–71. 22487979 10.1007/s12079-012-0158-2 PMC3368018

[19] Banerjee S, Dhar G, Haque I, et al. CCN5/WISP-2 expression in breast adenocarcinoma is associated with less frequent progression of the disease and suppresses the invasive phenotypes of tumor cells. Cancer Res. 2008;68(18):7606–12. 18794149 10.1158/0008-5472.CAN-08-1461

[20] Zhou G, Gui X, Qu W. Clinical significance of CCN5 and mutant p53 in primary and recurrent lesions of breast cancer. Am J Transl Res. 2021;13(7):8433–7. 34377340 PMC8340222

[21] Ferrand N, Stragier E, Redeuilh G, Sabbah M. Glucocorticoids induce CCN5/WISP-2 expression and attenuate invasion in oestrogen receptor-negative human breast cancer cells. Biochem J. 2012;447(1):71–9. 22765757 10.1042/BJ20120311

[22] Fritah A, Redeuilh G, Sabbah M. Molecular cloning and characterization of the human WISP-2/CCN5 gene promoter reveal its upregulation by oestrogens. J Endocrinol. 2006;191(3):613–24. 17170219 10.1677/joe.1.07009

[23] Banerjee SK, Maity G, Haque I, Ghosh A, Sarkar S, Gupta V, Campbell DR, Von Hoff D, Banerjee S. Human pancreatic cancer progression: an anarchy among CCN-siblings. J Cell Commun Signal. 2016;10(3):207–16. 27541366 10.1007/s12079-016-0343-9 PMC5055500

[24] Dhar G, Banerjee S, Dhar K, Tawfik O, Mayo MS, Vanveldhuizen PJ, Banerjee SK. Gain of oncogenic function of p53 mutants induces invasive phenotypes in human breast cancer cells by silencing CCN5/WISP-2. Cancer Res. 2008;68(12):4580–7. 18559502 10.1158/0008-5472.CAN-08-0316

[25] Haque I, Ghosh A, Acup S, Banerjee S, Dhar K, Ray A, Sarkar S, Kambhampati S, Banerjee SK. Leptin-induced ER-α-positive breast cancer cell viability and migration is mediated by suppressing CCN5-signaling via activating JAK/AKT/STAT-pathway. BMC Cancer. 2018;18(1):99. 29370782 10.1186/s12885-018-3993-6 PMC5785848

[26] Banerjee S, Saxena N, Sengupta K, et al. WISP-2 gene in human breast cancer: estrogen and progesterone inducible expression and regulation of tumor cell proliferation. Neoplasia. 2003;5(1):63–73. 12659671 10.1016/s1476-5586(03)80018-0 PMC1502127

[27] Fritah A, Saucier C, Wever OD, et al. Role of WISP-2/CCN5 in maintenance of a differentiated and noninvasive phenotype in human breast cancer cells. Mol Cell Biol. 2008;3:1114–23. 10.1128/MCB.01335-07 PMC2223394 18070926

[28] Talhouk A, McConechy MK, Leung S, et al. A clinically applicable molecular-based classification for endometrial cancers. Br J Cancer. 2015;113(2):299–310. 26172027 10.1038/bjc.2015.190 PMC4506381

[29] Kleer CG. Dual roles of CCN Proteins in breast cancer progression. J cell Commun Signal. 2006;10(3):217–22. 10.1007/s12079-016-0345-7 PMC5055502 27520547

[30] Ahmed KA, Hasib TA, Paul SK, et al. Potential role of CCN proteins in breast cancer: therapeutic advances and perspectives. Curr Oncol. 2021;28(6):4972–85. 34940056 10.3390/curroncol28060417 PMC8700172

[31] Sabbah M, Prunier C, Ferrand N, et al. CCN5, a novel transcriptional repressor of the transforming growth factor beta signaling pathway. Mol Cell Biol. 2011;31(7):1459–69. 21262769 10.1128/MCB.01316-10 PMC3135290

[32] Ji J, Jia S, Ji K, et al. Wnt1 inducible signaling pathway protein-2(WISP-2/CCN5):roles and regulation in human cancers(review). Oncol Rep. 2014;31(2):533–9. 24337439 10.3892/or.2013.2909

[33] Fuady JH, Bordoli MR, Abreu-Rodriguez I, Kristiansen G, Hoogewijs D, Stiehl DP, Wenger RH. Hypoxia-inducible factor-mediated induction of WISP-2 contributes to attenuated progression of breast cancer. Hypoxia. 2014;2:23–33. 27774464 10.2147/HP.S54404 PMC5045054

[34] Sarkar S, Ghosh A, Banerjee S, Maity G, Das A, Larson MA, Gupta V, Haque I, Tawfik O, Banerjee SK. CCN5/WISP-2 restores ER- proportional, variant in normal and neoplastic breast cells and sensitizes triple negative breast cancer cells to tamoxifen. Oncogene. 2017;6(5): e340. 10.1038/oncsis.2017.43 PMC5569333 28530705

[35] Mann S, Laucirica R, Carlson N, et al. Estrogen receptor beta expression in invasive breast cancer. Hum Pathol. 2001;32(1):113–8. 11172304 10.1053/hupa.2001.21506

[36] Rugo HS, Rumble RB, Macrae E, et al. Endocrine therapy for hormone receptor-positive metastatic breast cancer: American Society of Clinical Oncology guidelines. J Clin Oncol. 2016;34(25):3069–103. 27217461 10.1200/JCO.2016.67.1487

[37] Banerjee S, Sengupta K, Saxena NK, et al. Epidermal growth factor induces WISP-2/CCN5 expression in estrogen receptor-{alpha}-positive breast tumor cells through multiple molecular cross-talks. Mol Cancer Res. 2005;3(3):151–62. 15798095 10.1158/1541-7786.MCR-04-0130

[38] Dhar K, Banerjee S, Dhar G, et al. Insulin-like growth factor-1(IGF-1) induces WISP-2/CCN5 via multiple molecular cross-talks and is essential for mitogenic switch by IGF-1 axis in estrogen receptor-positive breast tumor cells. Cancer Res. 2007;67(4):1520–6. 17308090 10.1158/0008-5472.CAN-06-3753

[39] Mason HR, Grove-Strawser D, Rubin BS, et al. Estrogen induces CCN5 expression in the rat uterus in vivo. Endocrinology. 2004;145(2):976–82. 14605002 10.1210/en.2003-0823

[40] Davies SR, Watkins G, Mansel RE, et al. Differential expression and prognostic implications of the CCN family members WISP-2, WISP-2, and WISP-3 in human breast cancer. Ann Surg Oncol. 2007;14(6):1909–18. 17406949 10.1245/s10434-007-9376-x

