# Correction: Expression and clinical significance of CCN5 and the oestrogen receptor in advanced breast cancer

**DOI:** 10.1186/s12905-026-04638-1

**Published:** 2026-07-02

**Authors:** Guofeng Zhou, Wei Qu, Liu Yang, Aili Huang, Xingxing Gui

**Affiliations:** Department of Pathology, Nanchang People’S Hospital (the Third Hospital of Nanchang), No.1268, Jiuzhou Street, Xihu District, Nanchang, 330009 China

**Correction: BMC Women’s Health 25, 89 (2025)**

**https://doi.org/10.1186/s12905-025-03608-3**

In this article [1] the author name Xingxing Gui was incorrectly written as Xinxing Gui.

The original article has been corrected.
